# Dr. Robert A. Patchen

**Published:** 1903-10

**Authors:** 


Dr. Robert A. Patchen, of Des Moines, Ia., a graduate of the University of Buffalo, 1869, died at his home August 31, 1903, aged 53 years. Dr. Patchen occupied a prominent position as a physician and citizen in the city where he lived. He had been president of Polk County Medical Society, was a member of the Iowa State Medical Society, State Association of Railway Surgeons, and of the International Association of Railway Surgeons. The cause of his death, as reported, was an acute exacerbation of disease of the liver,—a malady that had afflicted him for a number of years.

